# A Probabilistic Model of the Economic Risk to Britain's Railway Network from Bridge Scour During Floods

**DOI:** 10.1111/risa.13370

**Published:** 2019-07-18

**Authors:** Rob Lamb, Paige Garside, Raghav Pant, Jim W. Hall

**Affiliations:** ^1^ JBA Trust Skipton North Yorkshire UK; ^2^ Lancaster Environment Centre Lancaster University, Bailrigg Lancaster UK; ^3^ Environmental Change Institute University of Oxford Oxford UK

**Keywords:** Bridge, flood risk, infrastructure, rail network, scour

## Abstract

Scour (localized erosion by water) is an important risk to bridges, and hence many infrastructure networks, around the world. In Britain, scour has caused the failure of railway bridges crossing rivers in more than 50 flood events. These events have been investigated in detail, providing a data set with which we develop and test a model to quantify scour risk. The risk analysis is formulated in terms of a generic, transferrable infrastructure network risk model. For some bridge failures, the severity of the causative flood was recorded or can be reconstructed. These data are combined with the background failure rate, and records of bridges that have not failed, to construct fragility curves that quantify the failure probability conditional on the severity of a flood event. The fragility curves generated are to some extent sensitive to the way in which these data are incorporated into the statistical analysis. The new fragility analysis is tested using flood events simulated from a spatial joint probability model for extreme river flows for all river gauging sites in Britain. The combined models appear robust in comparison with historical observations of the expected number of bridge failures in a flood event. The analysis is used to estimate the probability of single or multiple bridge failures in Britain's rail network. Combined with a model for passenger journey disruption in the event of bridge failure, we calculate a system‐wide estimate for the risk of scour failures in terms of passenger journey disruptions and associated economic costs.

## 1 INTRODUCTION

### 1.1 Bridge Scour

Scour is localized erosion by water that undermines bridge foundations, causing structural damage or collapse, with consequential safety risk and loss of utility for bridge users. It is cited as the most common cause of bridge failures in the United Kingdom (Kirby, Roca, Kitchen, Escarameia, & Chesterton, 2017) and the United States (Kattell & Eriksson, 1998). Bridge failures due to scour have occurred since the early years of the British railway network. Despite many risk mitigation measures (including codes of practice for design, construction, inspection, and maintenance), failures continue to happen occasionally, demonstrating that a residual risk exists. Scour risk can be assessed from multiple perspectives, including safety, economic, and reputational risks. This article is concerned with quantifying the residual risk in economic terms, primarily through the economic utility of passenger journeys. However, opportunities to include other important social and political aspects of risk relating to safety, loss of life, and public confidence in the railway network are also discussed.

On the railway network in Great Britain, there have been 100 recorded bridge failures at nontidal river crossings (up to 2013) attributed to scour caused by 54 flood events (Rail Safety and Standards Board, 2004; van Leeuwen & Lamb, 2014). We will use this historical information, combined with data about the railway network, passenger usage, and river flows, to estimate the risk of scour across the rail network in terms of loss of utility to rail passengers. The analysis is presented for a specific context relating to scour risk at British railway bridges, but is formulated in terms of a generic infrastructure network risk model that could be transferred to other networks and spatial weather‐related hazards.

### 1.2 Scour Risk Assessment

Scour risk is typically managed through the application of engineering standards and guidance, such as the U.K. Design Manual for Roads and Bridges (UK Roads Liaison Group, 2009), the U.S. National Bridge Inspection Standards (Federal Highway Administration, 2017a), and U.S. Forest Service scour assessment process (Kattell & Eriksson, 1998). Most scour management protocols are tiered, with initial, high‐level screening being applied to identify and prioritize bridges requiring more detailed assessment. Screening generally combines generic information about the bridge and watercourse, such as dimensions, bed material, and vegetation, and its scour history. The U.K. railway industry's scour assessments assign priority scores based on a ratio between foundation depth and estimated potential depth of scour within a prescribed modeling approach (Kirby et al., 2017). Further assessment considers a “design” flood event and may include more detailed site inspections, but is based on a scoring scheme rather than a fully probabilistic analysis.

Scour models have been proposed to predict equilibrium scour depths (Melville, 1997), time variation of scour (Hong, Goyal, Chiew, & Chua, 2012; Melville & Chiew, 1999), and scour at bridge abutments (Coleman, Lauchlan, & Melville, 2003). Most are functions of parameters describing the structure (pier or abutment shape, dimensions, and alignment), channel (cross‐section and roughness), water flow (depth and velocity), and sediment regime (cohesive or noncohesive). Despite extensive research, scour prediction involves many uncertainties (Lamb, Aspinall, Odbert, & Wagener, 2017; Zevenbergen, 2010) relating to the complexity of the physical processes and the difficulty (and cost) of collecting detailed asset‐specific data.

It is economically unfeasible to protect all bridges against all conceivable events; therefore, some residual risk has to be tolerated (UK Roads Liaison Group, 2009; Whitbread, Benn, & Hailes, 2000). The residual risk materializes in bridge failures that occasionally occur (e.g., flooding in 2015 caused damage to 235 road and foot bridges in Cumbria, northern England; Cumbria County Council, 2017). Here, we take a probabilistic approach to quantify scour risk at a broad scale, meaning that we consider risk over the whole rail network and taking account of the unpredictability of bridge failure events through data‐driven, statistical models.

We conceptualize risk as a function of a hazard, that is, a physical phenomenon carrying the potential to cause harm, and its consequences. The hazard that we consider is flooding, which subjects the bridge to hydraulic loading in various ways that may lead to scour, ultimately causing the bridge to fail. We define failure as total or partial collapse of a bridge such that it cannot convey traffic, described by a state variable, *s*, where *s* = 0 indicates the failed state. Any bridge not in a failed state is assumed to be operational (*s* = 1), although in general this assumption could be relaxed to allow for other damage states, and their consequences, to be considered.

Uncertainties about scour formation are reflected in the wide range of flood event magnitudes that can cause a bridge to fail, as shown in Table I and elsewhere (Flint, Fringer, Billington, Freyberg, & Diffenbaugh, 2017; Rail Safety and Standards Board, 2004; van Leeuwen & Lamb, 2014), and are accounted for here using a fragility function:

(1) $$F_{Y}\left(y\right)=\int_{0}^{y}f_{Y}\left(y\right)dy,$$

where the loading condition *Y* is treated as a random variable related to the severity of a flood event, which will be given a specific definition in Section 2.2, and *F_Y_*(*y*) is the cumulative probability distribution of the load at which failure occurs. The probability of failure conditional on *y* is Pr[*s* = 0|*y*] = *F_Y_*(*y*). It is not certain how often a bridge will experience flooding, nor how extreme those events might be; therefore, the load in any specific event will also be considered as a realization from a random variable, with distribution function:

(2) $$G_{Y}\left(y\right)=\int_{0}^{y}g_{Y}\left(y\right)dy.$$

**Table I:** Historical Rail Bridge Failure Events and Estimated Return Periods

Event	*N*	Day	Month	Year	Watercourse	Location	RP_est_ (Years)	RP_max_ (Years)
1	1	20	January	1846	River Medway	Between Tonbridge and Penshurst, Kent		23
2	1		February	1846	River Sheppey	Charlton Viaduct, near Shepton Mallet		25
3	9	29	September	1846	Eye Water, Tower Burn, Tyne	Grantshouse, Cockburnspath, East Lothian		35
4	2	8	July	1847	River Camel	Near Dunmmer Bridge, Bodmin, Cornwall		178
5	1	30	August	1866	River Esk	Between Grosmont and Whitby, Malton and Whitby Line		45
6	1	16	November	1866	River Aire	Apperley, West Yorkshire, Midland Railway Leeds to Lancaster Line	65	
7	2		February	1868	River Severn	Caersws, Central Wales Line		314
8	1	13	November	1869	River Tees	Darlington, County Durham, Merrybent Railway Company	13	
9	3	17	July	1880	Afon Wnion	Near Dolgellau, Bala, exact locations unknown		71
10	1		March	1881	Unnamed	Ladmanlow, near Buxton, Cromford and High Peak Railway		83
11	1		November	1882	Nant Burn	Near Taynuilt Station, Callander and Oban Railway		33
12	3	14	May	1886	River Teme	Near Bransford between Ludlow and Craven Arms		47
13	1	26	December	1886	River Rother	Selham, West Sussex, Midhurst Branch of London, Brighton and South Coast Railway		15
14	1		August	1891	Black Brook	Chorley, Lancashire, Chorley to Blackburn (Cherry Tree Line)		110
15	3	21	September	1891	Gala Water	Galashields	30	
16	1		August	1912	River Tas	Between Forncett and Flordon, Norfolk		27
16	1		August	1912	River Stiffkey	Fakenham, Norfolk		112
17	1	15	June	1914	Baddengorm Burn	Aviemore to Inverness, near Carrbridge Station, Highland Rlwy	1,000	
18	6	26	September	1915	Findhorn and Spey	Highland Railway	60	
19	4	8	July	1923	Bogbain Burn	Near Carrbridge	2,000	
20	1	9	June	1924	River Erewash	Pye Bridge, Ripley, Erewash Valley Line		42
21	3	23	July	1930	River Esk	Glaisdale, Esk Valley Line	1,000	
22	1	4	September	1931	River Esk	Glaisdale, Esk Valley Line	500	
23	1	21	June	1936	Mochdre Brook	Dulais Bridge, near Glandulais, Newtown, Powys	100	
24	1	7	September	1945	Llangollen Canal	Sun Bank Halt, GWR Llangollen Line		101
25	1		March	1947	River Wye	Strangford Viaduct, near Fawley, Hereford to Gloucester Line	100	
26	1	12	April	1947	Eastburn Beck	Eastburn Bridge, between Skipton and Keighley, Yorkshire		30
27	9	12	August	1948	River Eye	Harelawside Bridge, Smiddy Bridge, Mason's Bridge, Free Kirk Bridge, Eyemouth Viaduct and others between Dunbar and Berwick, East Coast main line	500	
27	1	12	August	1948	Birns Water	Between Humbie and Gifford, possibly Gilchriston.	500	
27	1	12	August	1948	Wooler Water	Haugh Head, Northumberland	200	
28	1	25	October	1949	Wooler Water	Wooler, Northumberland	111	
28	1	26	October	1949	Lilburn Burn	Near Lilburn Tower, Northumberland		23
29	1	19	November	1951	Midhurst Stream	Between Cocking and Midhurst, West Sussex	100	
30	1		October	1954	River Derwent	Bridge between Cockermouth and Workington		52
31	1	30	September	1960	River Creedy or Exe	Cowley Junction		43
32	1	12	December	1964	River Ystwyth	Llanilar, near Aberystwyth, Cambrian Railway	30	
32	1	12	December	1964	River Banwy	Castle Caereinion	30	
33	1	9	July	1968	River Chew	Viaduct near Pensford, Somerset		29
34	1	15	September	1968	River Wey	Between Farncombe and Godalming,	200	
34	1	15	September	1968	River Mole	Cobham	200	
34	1		September	1968	River Kennett	Nuns Wood, between Kennett and Higham, Newmarket	200	
34	1		September	1968	River Waveney	Bridge 317, Norwich Ipswich Main Line, Diss to Burston	200	
35	2	10	August	1969	Unknown (Lochaber)	Two bridges and six culverts washed out, exact locations n/a		16
36	1	31	August	1973	Glen Finnan	Fort William to Mallaig Line, bridge 313/051 Drumsallie		26
37	1		June	1985	River Deben	Whickham Market, East Suffolk Line		85
38	2	19	October	1987	R. Towy and R. Dulais	Glanrhyd and Llanwrda, Central Wales Line	50	
39	1	10	May	1988	Colne Brook	Wraysbury, bridge No. 71, Staines—Windsor Line		45
40	1	7	February	1989	River Ness	Near Inverness	100	
41	1	2	January	1991	Afon Twymyn	Cemmaes Road		226
42	3	14	January	1993	Rivers Tay, Earn, May	Dalguise, Forgandenny, Forteviot (SW of Perth)	100	
43	1		January	1994	River Severn	Cilcewydd, between Welshpool and Newtown		84
44	1		October	1997	Ettrick Water	Heatherlie Bridge near Selkirk		56
45	1	15	October	1998	Trib of R. Leven	Renton (Balloch), near Dunbarton		45
46	1		October	2000	River Taw	Weir Marsh Bridge		51
47	1	8	December	2000	River Exe	Cowley Junction, bridge carrying Barnstaple Branch		43
48	1	14	June	2002	River Irwell	Lower Ashenbottom Viaduct, Rawtenstall, Greater Manchester	100	
49	1	3	October	2002	River Tay	N/A		47
50	1		December	2002	Monks Brook	Between Eastleigh East and Romsey Junctions, Chandlers Ford		75
51	1	11	September	2003	River Rother	Beighton	2	
52	1	1	November	2006	Burn of Winless	Watten	15	
53	1	14	November	2009	River Crane	Feltham, West London	1	
54	1		December	2012	River Taw	Barnstaple line		51

*Notes*: For the September 1915 event on the Highland Railway, there is an account of 16 structures being washed away including bridges and other types of structure. In the absence of further evidence, the number of bridges involved in this event has been assumed to be scaled from the historical account according to the proportion of present‐day assets that are bridges, which is 31,663/79,830 ≈ 0.4, leading to an estimate of 16 × 0.4 ≈ 6 bridges, rounded to the nearest integer.

Event = index number of hydrological event associated with bridge failure(s); *N* = number of bridges failed during event; RP_est_ = estimated return period (years) of the associated flood based on historical analysis; RP_max_ = return period (years) of maximum flood estimated by interpolation from gauged river flow records.

### 1.3 Objectives and Outline

The goal of this article is to assess the risk to the railway network in Britain associated with bridge scour, and in doing so to develop and demonstrate the application of a generic, probabilistic infrastructure network risk model. A scour fragility function, *F_Y_*, is derived based on inferences from historical bridge failures. Rather than modeling scour processes explicitly, we interpret the historical failure events as primary observations. The proposed fragility function pools information from all rail bridges and historical failures for use in a “broad‐scale” network risk analysis, which is introduced in the next section.

The general formulation of the fragility function is then discussed, followed by a review of the bridge failure data. Next, the fragility function is fitted to the historical data by maximum likelihood estimation (MLE) and the results presented, with quantification of uncertainty. The proposed fragility function is combined with a spatial flood hazard model to estimate bridge failure rates over the railway networks, which are compared with the historical evidence to test the model. Finally, the probabilistic failure model is integrated with a passenger journey disruption model to estimate the network‐scale risk associated with bridge failures.

## 2 PROBABILISTIC RISK ANALYSIS FRAMEWORK

### 2.1 Broad‐Scale Risk Analysis

Risk is often quantified in terms of the expected value of losses incurred due to failure of an asset,

(3) $$\mu =\int_{y}z\left(s,y\right)F_{Y}\left(y\right)g_{Y}\left(y\right)dy,$$

where *z*(*s*, *y*) is a measure of loss as a function of asset state (assumed dependent on the load), which, in general, may encompass operational or economic consequences of asset failure; here a model of disruption to passenger journeys will be used. We will consider risk to bridges across the British railways, a national infrastructure network. This requires the flood hazard to be represented in terms of spatially coherent events, allowing for the possibility that multiple bridges in different locations could fail concurrently within an event. Although rare, such events have occurred in the past (see Table I) and may have significant impacts because failure at multiple locations limits possibilities for rerouting of trains, and may lead to the partial or complete breakdown of network functionality.

In a network of *D* bridges, contained in the set Δ, the load is considered as a (spatial) vector random variable **Y** = {*Y_k_*: *k* ∈ Δ}. For load event **Y** = **y**, the number of bridges that will fail is uncertain, with the uncertainty captured by the fragility function. Assuming failure processes at individual bridges to be independent (although realizations of bridge failures may be conditional upon spatially dependent loads), the number of failures expected in a particular event **y** is:

(4) $$\lambda_{|Y=y}=\sum_{k\in \Delta }\mathrm{Pr}\left[s_{k}=0|y_{k}\right]=\sum_{k\in \Delta }F_{Y,k}\left(y_{k}\right),$$

where *F_Y,k_*(*y_k_*) is a fragility function at the *k*th bridge.

The vector‐valued loads are now envisaged as being described by a multivariate probability density, *g* ***_Y_***(**y**). The expected number of failures, taking account of all flood hazard events, is:

(5) $$\lambda =\int \ldots \int_{R}\left[\sum_{k\in \Delta }F_{Y,k}\left(y_{k}\right)\right]g_{Y}\left(y\right)dy,$$

where ***R*** denotes a region of integration over all physically plausible loads.

The disposition of the network during or following an extreme flood can be represented as a vector of asset states **S** = {s*_k_*: *k* ∈ Δ}. The expected loss for any given network state is

(6) $$\mu \left(S\right)=z\left(S\right)\int \ldots \int_{R}\mathrm{Pr}\left[S\left|y\right.\right]g_{Y}\left(y\right)dy$$

and the network risk is the integration, over all possible loading, of the losses in all 2*^D^* − 1 failure states,

(7) $$\mu =\int \ldots \int_{R}\sum_{h=1}^{2^{D}-1}\left\{z\left(S_{h}\right)\mathrm{Pr}\left[S_{h}\left|y\right.\right]\right\}g_{Y}\left(y\right)dy,$$

where **S** *_h_* = {*s_i_*: *i* ∈ Δ}*_h_* is the *h*th network failure state considered (where a network failure is the failure of at least one bridge).

The loss function $z(S_{h})$ accounts for the location of bridge failure(s), the importance of the affected routes, and the potential for trains to be rerouted around the failure(s). In principle, these consequences of failure could be different for every failure state. For a national rail network with a large number of bridges, computing all 2*^D^* − 1 values of the loss function is an expensive task. In Section 6, we present a tractable way of approximating this calculation.

Few attempts have been made to derive risk models for bridge scour based on asset‐specific analysis of loading and failure probabilities. One study by Decò and Frangopol (2011) estimated annual failure probabilities at individual bridges, based on a method (Stein, Young, Trent, & Pearson, 1999) developed for use with U.S. National Bridge Inventory (NBI) (Federal Highway Administration, 2017b) data. Their risk assessment framework (Decò & Frangopol, 2011) was formulated as a multivariate analysis, similar in form to Equation (7), but accounting for independent, mutually exclusive hazards (such as scour, earthquake, or traffic loads) rather than spatially coherent events, as in our analysis. A difference is that our analysis explicitly models both the hazard and fragility using probabilistic models derived from observations of the hazard events (flooding) and bridge failures, whereas Deco and Frangopol's method involved empirical estimation of flow depths, combined with NBI scour vulnerability scores, to infer annual failure probabilities. In our analysis, the aim is not to assess scour risk at individual bridges, but rather for the whole rail network. We do this through pooling information spatially and temporally to estimate a scour fragility function for rail bridges in Britain, such that *F_Y_*_,_ *_k_*(*y_k_*) = *F_Y_*(*y_k_*), and then integrating the fragility function into a risk assessment based on Equation (7).

### 2.2 Generic Approach to Fragility Analysis and Definition of Loading Condition

Fragility functions for structures subjected to flood risk have usually been derived by geotechnical modeling, rather than empirical analysis of the performance of a population of assets during flood events (Buijs, Simm, Wallis, & Sayers, 2007; Hall et al., 2003; US Army Corps of Engineers, 1993; Van Gelder et al., 2008). In earthquake engineering, fragility functions have been derived statistically from observations of structures subjected to loads, such as peak ground acceleration or displacement (Federal Emergency Management Agency, 2012; Porter, Kennedy, & Bachman, 2007). In addition to failure observations, knowing which assets have survived extreme loading is useful because it can be inferred that the load required to cause failure is likely to be larger than any observed load. Observations of this form are treated as censored data (Kim & Feng, 2003; Klugman, Panjer, & Willmot, 2004; Shinozuka, Feng, Lee, & Naganuma, 2000) in fragility analysis.

Central to our study is the use of information from historical bridge failures. The data are derived from various sources. In some cases, even when quantitative measurements exist, there is ambiguity about how the evidence should be interpreted. For this reason, the loading condition cannot be defined precisely in terms of physical quantities that relate directly to scour, such as water depth or velocity.

Instead, the load is understood as a relative measure of the extremeness of a flood event, expressed in terms of its return period, *τ*, in years. This approach, which was identified as a feasible basis for a scour fragility analysis by an international expert group (Lamb et al., 2017), standardizes over river catchments of widely differing size (and hence characteristic flow rates). Other scales, such as annual exceedance probability (AEP), could be used to the same effect; return period was chosen because it is easily interpreted and matches the existing assessments of failure events.

The AEP of a flood approximates to 1/*τ*, and the load variable will be defined as $y=\tau ={\left(1-G_{X}\left(x\right)\right)}^{-1}$, where *G_X_*(*x*) is a model, in this case defined for annual probabilities, for the distribution of peak river flows represented as a random variable *X*. For some historical failures where the causative river flow cannot be estimated directly, the flood return period *y* has been inferred from other data, as described in the next section.

## 3 DATA

### 3.1 Railway Bridges in Britain

**Figure 1:**
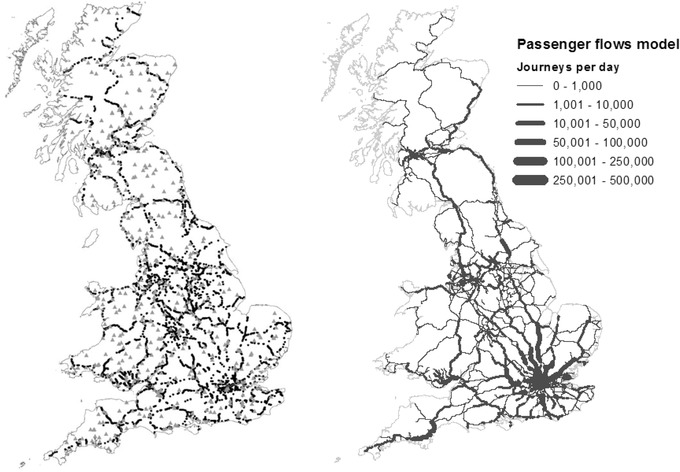
(a) Location of rail bridges over rivers and their floodplains (floodplain defined as areas potentially at risk of flooding with a 1/1,000 annual probability or more, not accounting for flood defenses), shown as dots. River gauges shown as gray triangles. (b) Estimated daily average number of passenger journeys on each edge of the rail network topological model described in Section 6.1.

Britain's railway infrastructure owner, Network Rail, maintains asset databases that the authors have consolidated using topographic data and aerial imagery to identify 8,877 bridges crossing rivers and their floodplains in Britain, shown in Fig. 1(a).

**Figure 2:**
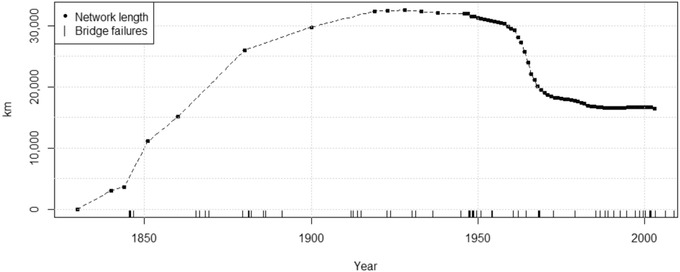
Reconstructed total length of railway network in Britain, 1830–2003 (inner ticks on horizontal axis are dates of bridge failures caused by scour; see Section 3.2).

The network has expanded and contracted over time, potentially leading to bias in estimates of failure probabilities if historical data were compared directly with the present‐day situation. Our data do not include bridge construction or decommissioning dates. However, some inferences may be drawn from a reconstruction of the total rail network length (Fig. 2) based on official information from the Department for Transport (HM Government, 2017b) for 1900 onward, and studies by Martí‐Henneberg (2013) and Haywood (2007) prior to 1900.

The mean network length over the period 1830–2003 is estimated as *T*_1830–2003_ *=* 22,291 km. The length in 2003 was *T*_2003_ = 16,493 km, which is taken to represent the present‐day network (the official value in 2012/2013 was 15,753 km, but figures after 2003 were omitted because of a change in calculation methodology in 2004). These estimates will be used in the fragility analysis.

### 3.2 Historical Bridge Failures Attributed to Scour

Previous studies (Rail Safety and Standards Board, 2004; van Leeuwen & Lamb, 2014) catalogued 138 railway bridge failures caused by scour in Britain between 1846 and 2013. After removing estuary crossings (where scour process may have different physical drivers) and one misclassification, we found 100 failures associated with 54 flood events, which are summarized in Fig. 2 and Table I.

For 51 of the failures, there are estimates of the return period of the associated hydrological event (labeled RP_est_ in Table I) from detailed assessments of historical rainfall and river flow information, previous research, published studies of notable floods (Black & Law, 2004), and interpretations of contemporary newspaper reports.

Of the 54 events that have caused bridge failures, 35 (65%) are in the autumn, winter, and spring months of September–March (inclusive). These figures are consistent with studies of flood seasonality in Britain, which show that the highest river flow rates (defined either as annual maxima or extreme threshold exceedances) occur predominantly in September–March (Cunderlik, Ouarda, & Bobée, 2004; Hall & Blöschl, 2018). There is also a nonnegligible chance of flood flows occurring in the summer (Cunderlik et al., 2004), and for some rivers the most extreme flows have occurred during the summer months (Black & Werritty, 1997), while recent history has demonstrated that severe flooding can happen in summer, for example, in 2007 when Britain experienced one of the most damaging flood episodes in modern history, including riverine flooding, during the months of June and July. Table I contains 16 events (29%) linked to railway bridge failures in the months of June–August, which suggests a higher prevalence of failure events in these months than might be expected if failures were a deterministic function of river flow, reinforcing our motivations for modeling bridge failure probabilistically.

Some flood events have caused more than one bridge to fail. The potential for co‐occurrence of bridge failures within the same hydrological event does not affect our fragility analysis in Section 4, in which every bridge failure is included individually. However, it is accounted for in our risk estimation (Sections 5 and 6), where load events are simulated from a model that captures the spatial and temporal dependence of river flows in Britain, conditioned on long‐term observations.

### 3.3 Hydrological Analysis

There are 49 failures associated with 34 flood events that lack detailed return period assessments. For these events, and for the many bridges that have not failed, an estimate of the most extreme load experienced at each bridge location will be used in a censored maximum likelihood (ML) estimate of the fragility function parameters (Section 4).

River flow records were obtained for 494 gauges from the U.K. National River Flow Archive (Centre for Ecology and Hydrology, 2017) for the years 1960–2008, providing flow archive data for 48% of the failure events. A generalized extreme value distribution was fitted to annual maximum river flows at each gauge to enable estimation of return periods. The return period, *τ_k_*, of the flow at bridge *k* was estimated using five neighboring gauges by inverse distance‐weighted interpolation, such that:

(8) $$\tau_{k}=\frac{\sum_{i=1}^{i=5}\tau_{i}/d_{i}}{\sum_{i=1}^{i=5}1/d_{i}},$$

where *τ_i_* is the return period of the flow at the *i*th nearest gauge, and the proximity of gauges was assessed in terms of *d_i_*, the distance between the centroids of the upstream catchment area draining to gauge *i* and the centroid of the catchment area draining to the bridge *k*.

With this interpolation procedure, information in small river basins can be contributed from nearby gauging stations on different watercourses, allowing for situations when one storm affects multiple gauges. For large basins, gauges on the same branch of the river network contribute more, reflecting the importance of river routing. It has previously been applied (Lamb et al., 2010) and tested (Environment Agency, 2011) in a similar context for flood risk modeling. Data transfers of this type, based on distance between river basin centroids, have been found to perform well for estimating annual maximum flows on rivers in the United Kingdom (Kjeldsen & Jones, 2010; Kjeldsen, Jones, & Morris, 2014).

River flow data from 1960 to 2008 have been interpreted as representative of conditions during the lifetime of each bridge, an assumption that may underestimate the true maximum load for bridges that have been in service for longer. This approximation has been tolerated because its influence on the fragility function likelihood (Section 4) is small relative to the large difference between the number of failed and nonfailed bridges.

## 4 FRAGILITY ANALYSIS

### 4.1 Choice of Fragility Function

Our proposed fragility function is a lognormal distribution, such that the loading condition associated with a bridge failure is modeled as

(9) $$\mathrm{Pr}\left(s=0|Y=y\right)=F_{Y}\left(y\right)=\Phi \left(\frac{\ln\left(y/\theta \right)}{\beta }\right),$$

where Φ(·) is the standard normal cdf, *θ* is a location parameter, and *β* is a dispersion parameter, and

(10) $$F_{Y}\left(y\right)=\int_{y}f_{Y}\left(y\right)dy=\int_{y}\varphi \left(\frac{\ln\left(y/\theta \right)}{\beta }\right),$$

where ϕ(·) is the standard normal pdf.

A lognormal fragility function was chosen because it is a parsimonious two‐parameter distribution with positive support (ensuring that unrealistic negative loads cannot occur), and with many precedents for its use in fragility analysis (Porter et al., 2007). This does not necessarily mean that a lognormal distribution is an appropriate fragility function, but our results do not suggest any reason to choose a different distribution.

### 4.2 Maximum Likelihood Estimation

The parameters *θ* and *β* are unknown and must be estimated; we use an ML approach. The bridge failures are treated as independent observations and all failure events are pooled into one large sample, removing any explicit consideration of time. Hence, the replacement of failed bridges is not modeled. It is reasonable to assume that most bridges will have been repaired, and the influence of individual failures compared with the (much larger) total number of bridges on the network is negligible.

The data fall into three sets of observations that will contribute information to the analysis, labeled A, B, and C, described in Table II.

**Table II:** Partition of Bridge Observations into Three Sets

Set A	Historical bridge failures with associated flood event return periods, which are regarded as known values for the loading condition at failure. The likelihood for the failure observations that have known flood return periods is the density, *f_Y_*(*y*).
Set B	Historical bridge failures associated with an unknown flood return period are incorporated as a form of left‐censored data, for which the likelihood is *F_Y_*(*y*), the probability of loads not exceeding the estimated maximum historical load derived from gauged flood flows.
Set C	Bridges that are assumed not to have failed (“survivor” bridges), for which the likelihood is 1 − *F_Y_*(*y*), which is the probability of exceeding the estimated maximum historical loading derived from the gauged flows.

The likelihood function is:

(11) $$\begin{array}{ccc} L\left(\Theta \right) & = & \prod_{i\in \Delta }{\left\{f\left(y_{i};\Theta \right)\right\}}^{\delta_{A}\left(i\right)}{\left\{F\left(y_{i};\Theta \right)\right\}}^{\delta_{B}\left(i\right)}\\ & & {\left\{1-F\left(y_{i};\Theta \right)\right\}}^{\delta_{C}\left(i\right)}, \end{array}$$

where the indicator variable *δ*(*i*) takes the values:

*δ_A_*(*i*) = 1; *δ_B_*(*i*) = 0; *δ_C_*(*i*) = 0 for a bridge failure with known load (set A),
*δ_A_*(*i*) = 0; *δ_B_*(*i*) = 1; *δ_C_*(*i*) = 0 for a bridge failure with unknown load (set B),
*δ_A_*(*i*) = 0; *δ_B_*(*i*) = 0; *δ_C_*(*i*) = 1 for a surviving bridge (set C).

The parameters are Θ = {*θ*, *β*}, and Δ is the set of observations of bridge states, combining all bridge failures and surviving bridges.

The three contributions to the likelihood are now described in more detail.

#### 4.2.1 Contribution from Bridge Failures with Known Load (Set A)

The likelihood for a failure observation associated with load *y* is:

(12) $$L_{\theta ,\beta }\left(y\right)=\varphi \left(\frac{\ln\left(y/\theta \right)}{\beta }\right).$$

Equation (12) includes the contribution to the likelihood from the set of bridge failures where the failure event load *y* has been assessed. The likelihood of observing this set of historical failures with associated loads **y** = {*y_i_*: *i* ∈ A) is:

(13) $$L_{A}=\prod_{i\in A}\varphi \left(\frac{\ln\left(y_{i}/\theta \right)}{\beta }\right).$$

#### 4.2.2 Contribution from Bridge Failures with Unknown Load (Set B)

The unknown failure load is assumed to have been no greater than y_i_*, the estimated maximum historical load at each bridge. The contribution to the likelihood for *i* ∈ B is then:

(14) $$L_{B}=\prod_{i\in B}\Phi \left(\frac{\ln\left(y_{i}^{*}/\theta \right)}{\beta }\right).$$

#### 4.2.3 Contribution from Surviving Bridges (Set C)

Any surviving bridge, *i* ∈ C, is known to have resisted loads as large as y_i_*, so the likelihood of the observation is Pr[*Y* > y_i_*], or

(15) $$L_{\theta ,\beta }\left(y_{i}^{*}\right)=1-\Phi \left(\frac{\ln\left(y_{i}^{*}/\theta \right)}{\beta }\right).$$

The contribution from the observations of surviving bridges in set C is therefore:

(16) $$L_{C}=\prod_{i\in C}\left[1-\Phi \left(\frac{\ln\left(y_{i}^{*}/\theta \right)}{\beta }\right)\right].$$

The bridges in set C represent the present‐day situation but, as discussed earlier, the network has on average been more extensive in the past, with more bridges than today. This means that *L_c_* may underestimate the probability of observing a survivor bridge when considered alongside historical failure data. Assuming the average number of bridges per unit network length has remained constant, the ratio

(17) $$\gamma =T_{1830-2003}/T_{2003}\approx 1.35$$

is applied as an adjustment to inflate the likelihood associated with survivor bridges, reflecting the average extent of the historical network relative to the present day.

#### 4.2.4 Maximum Likelihood Analysis

Combining Equations (13), (14), (16), and (17), the function to be maximized is the log‐likelihood:

(18) $$\begin{array}{ccc} \ln(L) & = & \sum_{i\in A}\ln\varphi \left(\frac{\ln\left(y_{i}/\theta \right)}{\beta }\right)+\sum_{i\in B}\ln\Phi \left(\frac{\ln\left(y_{i}^{*}/\theta \right)}{\beta }\right)\\ & & +\;\gamma \sum_{i\in C}\ln\left[1-\Phi \left(\frac{\ln\left(y_{i}^{*}/\theta \right)}{\beta }\right)\right]. \end{array}$$

Equation (18) was maximized in three stages. First the likelihood was evaluated on a wide trial grid of 250,000 values of *θ* and *β*. The grid was then progressively refined to focus on the region of ML so as to enclose the 95% confidence region (see below). Finally, a Nelder–Mead (1965) optimization procedure was applied to obtain the ML estimate.

**Figure 3:**
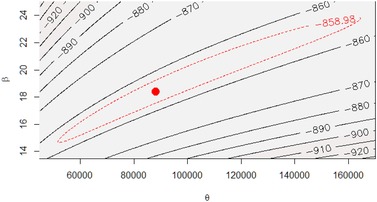
Likelihood surface contours conditioned on observations in sets A, B, and C. Red dot is maximum; dashed line encloses 95% confidence region.

Fig. 3 shows the log‐likelihood surface, conditioned on all available data in sets A, B, and C. The 95% confidence region, plotted as a dotted line in Fig. 3, satisfies the condition (Clarke, 1994; McCullagh & Nelder, 1989):

(19) $$2\left\{\ln L\left(\hat{\theta },\hat{\beta }\right)-\ln L\left(\theta ,\beta \right)\right\}\leq \chi_{\alpha }^{2},$$

where $\hat{\theta }$ and $\hat{\beta }$ are the ML estimates and *α* = 0.05 is the confidence level.

The estimation procedure was repeated, conditioning each time on different combinations of the data sets A, B, and C to explore the influence of progressively introducing information from each set of observations. The ML parameter estimates are shown in Table III.

**Table III:** Estimated Fragility Curve Parameters

Conditioning Data Type	Data Sets	Notes	Location, *θ*	Dispersion, *β*
Failed bridges	A	Failures with estimated flood RP based on detailed assessment	145	5.1
Failed bridges	A, B	As for set A plus failures with estimated maximum historical flood RP from gauged data	35	8.1
Failed and surviving bridges	A, B, C	All failure and survivor observations	88,239	18.4

**Figure 4:**
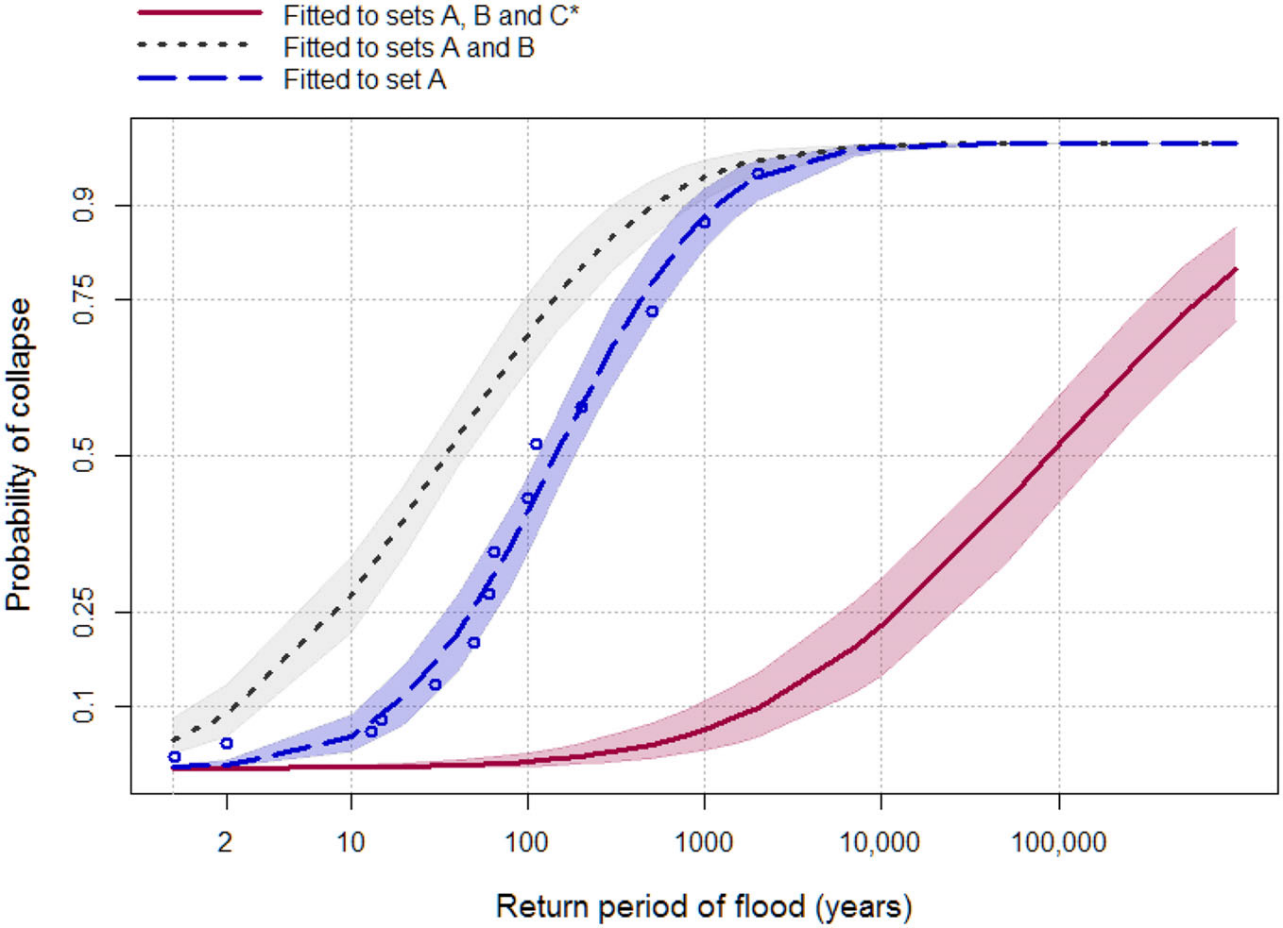
Fragility curves with shaded confidence regions. Curves fitted by MLE to historical bridge failure observations with an assessed flood return period (set A), all historical bridge failures (sets A and B), and all available data including surviving bridges (sets A, B, and C). Weibull plotting positions are shown for the observations in set A.

Fig. 4 shows the resulting family of fragility curves. The dots are the empirical distribution of failure observations in set A, with failure probabilities estimated from the rank, *r*, of the associated flood event return periods according to the Weibull plotting position (*r*/*N* + 1). The curves conditioned on sets A and B take into account only the subset of bridges known to have failed. As expected, failure probabilities increase when the additional failure observations in set B are introduced, but the dispersion also increases, reflecting uncertainty about the severity of flood events experienced at those bridges.

The solid curve fitted to all observations (sets A, B, and C) accounts for all bridges and is the proposed fragility model. As expected, it indicates a much greater resilience than the curves fitted to the failure observations alone, reflecting the fact that many more bridges have survived than have failed.

Our fragility analysis allows us to quantify and constrain uncertainties about scour failure probabilities based on observations of past bridge failures. The unpredictable nature of failure events means that quantification of these uncertainties is important, but the complexity of the processes and relative rarity of failures makes this difficult. A recent study that pooled expert assessments of bridge failure probabilities revealed very wide uncertainties (Lamb et al., 2017) when considering generic classes of bridge and watercourse type. The fragility function we have derived in this article sits within the uncertainty bounds elicited from expert judgments, as shown in Fig. 3 in the work cited above (Lamb et al., 2017). By making inferences from observed failure events, in this case at railway bridges, the uncertainty surrounding our failure probability analysis has been reduced by up to an order of magnitude compared with the experts’ judgments.

## 5 SIMULATED BRIDGE FAILURES

### 5.1 Spatial Load Event Model

Given a model of the joint (spatial) distribution of flood events over the network, *g* **_Y_**(**y**), Equation (7) can be applied to estimate the expected number of bridge failures per event. It is hard to specify *g_Y_*(**y**) directly. Instead, we use Monte Carlo simulation from a model for spatially coherent extreme river flows introduced by Lamb et al. (2010) and Keef, Tawn, and Lamb (2013), based on theory developed by Heffernan and Tawn (2004). The theory provides an asymptotically justified model for the conditional distribution of a set of variables, given that one variable exceeds a threshold. This conditional analysis allows for extrapolation into the joint tail region of the data, and hence can be used to simulate events more extreme than any previously observed. The model was fitted to data from the 494 river flow gauges described earlier, and has previously been applied in different forms to support U.K. government assessments of national flood risk (HM Government, 2016; Wood et al., 2016).

### 5.2 Probability Distribution of Concurrent Bridge Failures

Since there are the 100 bridge failures in Table I associated with 54 flood events, the mean number of failures in any event where at least one failure occurs is estimated to be *λ*_|_ *_n_*_>0_ = 100/54 ≈ 1.85. A 95% confidence interval around this estimate gives *λ*_|_ *_n_*_>0_ ± 1.96 *s* = (1.34, 2.38), where *s* = σ_A∪B_/54^(1/2)^ = 0.23 is the standard error of the mean, and σ_A∪B_ the standard deviation of the number of bridge failures observed in each event.

For comparison, a Monte Carlo simulation procedure described by Keef et al. (2013) was used to generate 1,000 samples, each comprising 54 load events, **y** *^j^* (*j =* 1,*…*, 54), conceptually equivalent to random samples drawn from the joint distribution *g* **_Y_**(**y**). The data were interpolated to railway bridge locations using Equation (8).

For any simulated event *j*, the probability of encountering one or more bridge failures is

(20) $$\mathrm{Pr}\left[n^{j}>0\right]=1-\left[\prod_{k\in \Delta }\left\{1-F_{Y}\left(y_{k}^{j}\right)\right\}\right]$$

and the expected number of failures, conditional on encountering at least one failure per sampled event, is

(21) $$\begin{array}{c} {\hat{\lambda }}_{|n>0}\approx \gamma \sum_{j=1}^{54}\left[\sum_{k\in \Delta }F_{Y}\left(y_{k}^{j}\right)\mathrm{Pr}\left[Y_{k}^{j}=y_{k}^{j}\left|n^{j}>0\right.\right]\right], \end{array}$$

where

(22) $$\mathrm{Pr}\left[Y_{k}^{j}=y_{k}^{j}\left|n^{j}>0\right.\right]=\frac{\mathrm{Pr}\left[n^{j}>0\right]}{\sum_{j}\mathrm{Pr}\left[n^{j}>0\right]}$$

and *γ* adjusts for the historical evolution of the network, as before.

**Figure 5:**
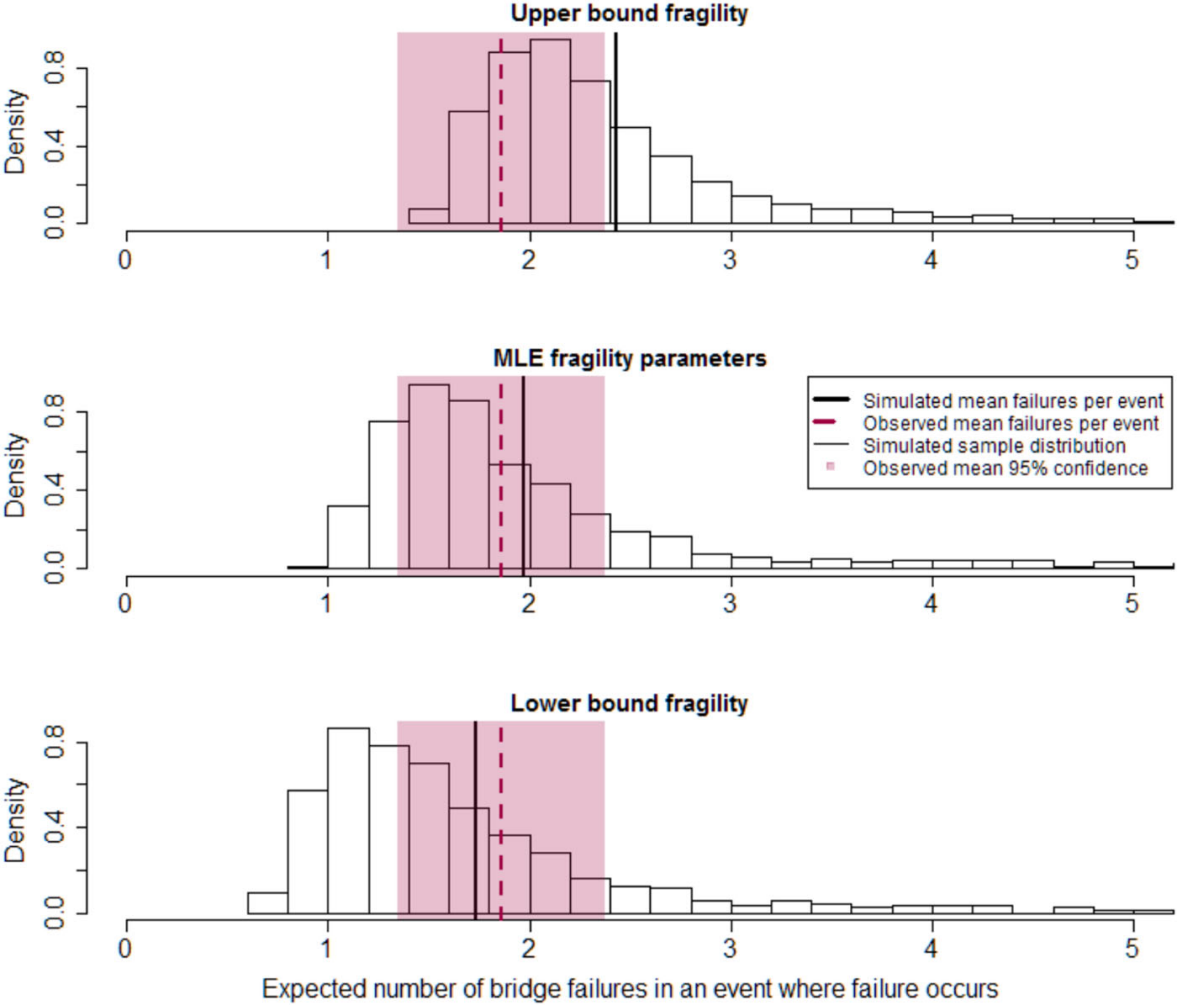
Expected number of bridge failures in a spatial load event. Dashed line is the mean number of failures in 54 observed events, with shaded 95% confidence interval. Histogram data are expected number of failures in samples of 54 simulated load events, with conditional failure probabilities computed from a fragility curve conditioned on all observations (sets “A, B, and C” in Fig. 4). Lower and upper bounds reflect the 95% confidence region around the fragility function (shaded region in Fig. 4).

The conditional expectation, Equation (21), provides a statistic derived from modeled data that can be compared with the observed failure events. This modeled expected failure rate, $\hat{\lambda }$, is compared with *λ* in Fig. 5, using the fragility function conditioned on observations in sets A, B, and C. Histograms in Fig. 5 show the distribution of $\hat{\lambda }$ over the 1,000 trials. The solid black line is the central (mean) estimate. The middle panel shows results for the MLE fragility parameters (*θ* = 88,239, *β* = 18.4). The lower and upper bound plots correspond to the 95% confidence region around the fragility function (shaded area surrounding the solid curve in Fig. 4). The dashed line in each panel of Fig. 5 is the observed mean, *λ*, with its 95% confidence region indicated as a shaded area.

With the MLE fragility parameters, the modeled data appear to overpredict slightly the number of bridge failures expected in an event, although the modeled central estimate $\hat{\lambda }$ = 1.97 lies within the 95% confidence interval around the observed value *λ* = 1.85. The mean failure counts associated with the upper and lower bounds of the fragility function confidence region enclose the observed mean.

## 6 NETWORK RISK ASSESSMENT

### 6.1 Passenger Journey Disruption Model

The network risk of bridge failure, *μ*, can be assessed using Equation (7) given a suitable loss function, *z*(**S**). We use a model (Pant, Hall, & Blainey, 2016) of the number of passenger journeys disrupted in a 24‐hour period if one or more bridges fail somewhere in the network. By expressing losses in terms of the daily disruption rate, we can integrate over flood event and bridge failure probabilities independently of any assumptions about how long disruption may persist in the event of a bridge failure, which will be considered later.

The model uses two data sources:

- Time table data from train operating companies (Association of Train Operating Companies, 2013b), giving weekly schedules detailing the stations on the network where each train calls. This information is used to estimate the number of trains running along different routes of the network on a typical day.
- Station usage statistics from the Office of Rail and Road (Office of Rail Regulation, 2013), which record annual numbers of passengers entering, exiting, and interchanging journeys at all stations in the network. This information is converted into daily estimates of station usage.

The model estimates the aggregated daily numbers of journeys distributed along different routes from each station. This journey distribution is a function of the number of trains and the volume of journeys (passenger entries, exits, and interchanges) along stations on each route. Once the aggregated daily journey numbers are distributed along different routes from each station, origin–destination (O–D) estimates are made for exit stops along each route by assuming O–D values will be in proportion to the volume of journeys attracted at exit stations. By repeating the above steps for all stations, the model generates a comprehensive O–D journey assignment for the entire network.

To estimate disruption for any failure state, the model is developed further to estimate the spatial distribution of journey reductions associated with a failure on any edge in the network topology, which is assumed to cause full closure of the affected part of the network. The model finds the number of O–D journeys along the affected section and searches for alternative routes (based on shortest distance) to complete these journeys. If there are no alternative routes in the residual network, then the number of journeys lost equals the O–D flows through the affected section. If alternative routes exist, some journeys are still lost as passengers might not travel if the alternative journey is longer than the original one. The railway passenger demand forecasting handbook (Association of Train Operating Companies, 2013a) provides estimates for the decay of journeys with increasing distances, which are used in the model. By looking at all disrupted routes, the model estimates the passenger trips lost over the entire network (on a per‐day basis), which is adopted as a loss function on the assumption that the closure of the routes is caused by bridge failures. In Section 7.1, we extend this analysis to consider the duration of disruption to passenger journeys, and consequential economic loss.

### 6.2 Network Edge Failure States

The network topology of the passenger journey disruption model contains 2,047 edges, which we denote by the set Λ. To compute network disruption, we map the 8,877 bridges onto the 2,047 network edges, so that bridge failure probabilities calculated using the spatial flood event model and the fragility curve can be translated into probabilities of failure (and hence passenger disruption) occurring on a given edge. For an edge, *E*, passing over a subset of bridges, **B** ⊂ Δ, the vector **S** *_B_* = {*s_k_*: *k* ∈ **B**) describes the state of each bridge. If one or more of the bridges crossed by an edge fails, then the edge is considered to be in a “failed state.” We describe this situation by writing *s^E^* = 0, where the superscript notation is used to denote the failure of an edge, *E*, rather than a bridge. The probability of such an event is:

(23) $$\mathrm{Pr}\left[s^{E}=0|y\right]=1-\prod_{k\in B}\left(1-\mathrm{Pr}\left[s_{k}=0|y_{k}\right]\right),$$

where Pr[*s_k_* = 0|*y_k_*] is the probability of failure of the *k*th bridge crossed by the edge, which is evaluated using the fragility function, conditional on a load supplied by the spatial river flow model.

An *n*th‐order failure state refers to an event in which there is a failure of each of a set of *n* (*n* > 0) edges, **E** = {*E_i_*: *i* ⊂ Λ, |**E**| = *n*}. The conditional probability of the failure state is given by combining the probabilities of states on the individual edges,

(24) $$\mathrm{Pr}\left[s^{E}=0|y\right]=\prod_{i\in \Lambda }\mathrm{Pr}[s^{i}|y].$$

The set of possible network failure states is the power set ℘*_≥_*_1_(Λ). Using Equation (24), the expected loss integrated over all flood events and network edge failure states is:

(25) $$\begin{array}{c} \mu =\int \ldots \int_{R}\sum_{E\in {℘}_{\geq 1}\left(\Lambda \right)}\left\{z\left(s^{E}\right)\mathrm{Pr}\left[s^{E}=0\left|y\right.\right]\right\}g_{Y}\left(y\right)dy, \end{array}$$

which is analogous to Equation (7), but now expressed in terms of network edge failures, for which the passenger journey disruption model provides a suitable loss function.

### 6.3 Risk Integration

Equation (25) represents the integration of expected disruption over the distribution of load events. We take a Monte Carlo approach to approximate the integral by averaging estimates of expected network disruption over a large set of stochastically simulated flood events, representing 10,000 years of simulated data. This sample is generated from the spatial river flow model (Section 5.1), which simulates events at an annual rate inferred from the observed river flow data, in this case 4.3 events/year.

The analysis of complex infrastructure networks that involve many components and multiple damage states is computationally demanding. A general strategy is to prioritize important states, which can be achieved using a probability sort algorithm (van Erp, Linger, Khakzad, & van Gelder, 2017), even for very large networks. Equation (25) requires the evaluation of all 2^|Λ|^ − 1 ≈ *O*(10^2,672^) possible network edge failure states, which is not feasible. However, many states will make a negligible contribution to the risk: some, particularly higher‐order failure combinations, because their probability will be negligible, and others because they may cause relatively little disruption. Therefore, we approximate *μ* by evaluating a subset of network states, Ψ (|Ψ| ≪ 2^|Λ|^ − 1), starting with the first‐order states, which have the highest probabilities, and then identifying those higher‐order states that would cause the greatest disruption, such that

(26) $$\mu \approx \mu \left(\Psi \right)=\frac{1}{43,\;000}\sum_{j=1}^{43,000}{\bar{z}}^{j}\left(\Psi \right),$$

where

(27) $${\bar{z}}^{j}\left(\Psi \right)=\sum_{E\in \Psi }\left\{z\left(s^{E}\right)\mathrm{Pr}\left[s^{E}=0\left|y^{j}\right.\right]\right\}$$

is the daily number of journeys expected to suffer disruption in the *j*th simulated spatial flood event, estimated from the network disruption of all evaluated network failure states weighted by the associated failure probabilities.

**Figure 6:**
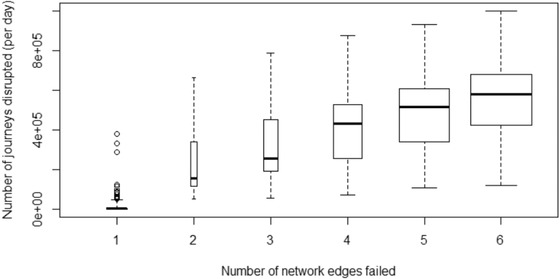
Passenger journeys disrupted (daily rate) for failure of one network edge or combinations of up to six network edges. Thick horizontal line is the mean, boxes span the interquartile range, area of box is proportional to number of failure states, circles represent the 19 most important first‐order failures, which were used to compute disruption for a subset of higher‐order failure states.

The subset Ψ was chosen by first identifying edges on which a bridge failure would cause disruption of more than 50,000 journeys per day. There are 19 such edges. The disruption arising from failure combinations of up to six of these 19 edges was then also calculated, meaning that Equations (26) and (27) were evaluated for the set Ψ containing all 2,047 first‐order edge failures plus 34,370 of the most important higher‐order failure cases. The practical constraint on evaluating further failure scenarios is the inclusion of the journey rerouting algorithm, which represents the adaptive capacity of the network, and was prohibitively expensive to evaluate beyond the sixth‐order failure states. The number of journeys predicted to be disrupted by all failure states within Ψ, expressed as an estimated daily rate, is shown in Fig. 6. The mean increases sharply over the first‐ to fourth‐order states, but grows at a much smaller rate for the higher‐order states, suggesting that there is unlikely to be a significant error introduced by truncating the analysis at the sixth‐order failure states.

### 6.4 Results

**Figure 7:**
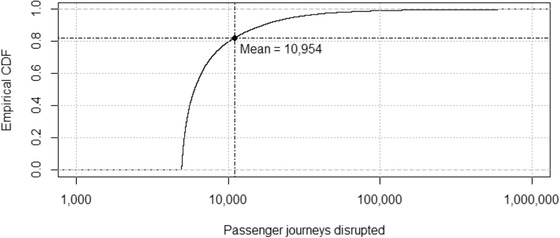
Empirical cumulative distribution of ${\bar{z}}^{j}$, the daily disruption to passenger journeys on the rail network estimated from 43,000 simulated spatial flood events.

The empirical distribution of the expected number of passenger journeys disrupted in the simulated spatial flood events is plotted in Fig. 7. The distribution is highly skewed, with most flood events expected to cause little disruption, but a long tail of extreme floods contributing significantly to the risk, emphasizing the need to model such low‐probability, high‐consequence events explicitly. Averaging over all simulated floods, the expected number of disrupted passenger journeys per event is *μ* = 10,954 per day of disruption.

**Figure 8:**
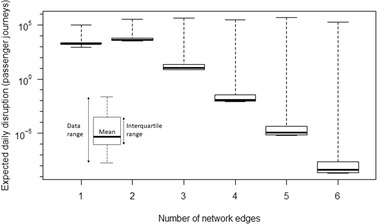
Contribution to risk in terms of the daily expected rate of passenger journey disruption estimated from 43,000 simulated flood events, assessed separately for first‐ to sixth‐order failure states.

The contributions to risk for the first‐ to sixth‐order failure states are further explored in Fig. 8, where the boxplots represent the distribution of daily expected rate of passenger journey disruption over all 43,000 simulated flood events (note the logarithmic scale and the presence of a very small number of important failure states, visible as “whiskers,” that contribute disproportionately to the total risk). Table IV summarizes the data plotted in Fig. 8. Averaging over all flood events, the daily rate of passenger disruption attributable to the failure of bridge(s) on a single network edge is expected to be 2,682 (24% of the total risk), while that attributable to failures in combinations of at least two edges is estimated to be 8,272 (76% of the risk).

**Table IV:** Summary Statistics of the Contribution to Risk in Terms of the Daily Rate of Passenger Journeys Expected to Suffer Disruption When Assessed Over 43,000 Simulated Flood Events and Failure States for All First‐ to Sixth‐Order Failure States Evaluated

		Number of network edges failed					
		1	2	3	4	5	≥6
Number of passenger journeys expected to suffer disruption	Min.	849	3,253	7	<1	<1	<1
	Median	1,812	4,113	10	<1	<1	<1
	Mean	2,682	7,773	323	94	65	17
	Max.	102,860	313,334	426,883	303,543	491,528	168,839

## 7 DISCUSSION

### 7.1 Quantification of Economic Risk

In the U.K. railway industry, the operational consequences of scour risk are realized through a complex internal business model. Costs of disruption are transferred between multiple organizations via a system of compensation payments, which are based on delays incurred by rail users. Further costs are associated with emergency works, monitoring, maintenance, and capital expenditure on resilience. Our analysis does not include these impacts, which would require additional proprietary data and models.

However, the economic impacts of scour risk can be assessed in part by considering the loss of utility associated with disruption to passenger journeys. Our model estimates journey disruption at a daily time scale, whereas the loss of utility caused by a bridge failure will also depend on the time for which the disruption persists. Neglecting the effect of demand transfer to other modes of transport (but accounting for the rerouting of journeys within the rail network itself; see Section 6), we will approximate the total journey disruption caused by a failure event as the daily disruption rate multiplied by an estimate of the time required to reinstate a bridge, informed by actual events as discussed below.

Following a bridge failure and consequent line closure, the time needed to reopen the bridge depends on many unpredictable factors, including constraints imposed by continuing weather events, site access and geotechnical issues, workforce resources, and competing emergency funding priorities. Our estimate of a mean disruption duration is informed by four scour failure events in the United Kingdom and Ireland since 1987, and for which accident reports have been published, listed in Table V.

**Table V:** Documented Scour‐Related Bridge Failures and Reinstatement Times

Location and Date of Failure	Cause of Failure	Days Until Reopened	Source
Lammington, Scotland, December 31, 2015	Pier partially undermined by scour, leading to sudden movement of the viaduct deck	54	Rail Accident Investigation Branch, Department for Transport, Report 22/2016, November 2016, http://www.raib.gov.uk
Feltham, England, November 14, 2009	Abutment undermined by scour, linked to obstruction of arch, causing subsidence and dislocation of arch ring	180 (estimated, date bridge fully reinstated, reported as middle of month)	Rail Accident Investigation Branch, Department for Transport, Report 17/2010, September 2010, http://www.raib.gov.uk
Malahide, Ireland, August 21, 2009	Collapse caused by scour following deterioration of protection weir	87	Railway Accident Investigation Unit, Investigation Report No. 2010—R004, August 2010
Glanrhyd, Wales, October 19, 1987	Scour failure of piers resulting in collapse of the bridge superstructure	377	Department of Transport Accident Report, HMSO, 1990, ISBN 0 11 550961 5

The average length of disruption in these cases was 174 days. Adopting this figure as a general estimate and combining with our disruption risk model, the expected disruption per failure event is 174 × 10,954 = 1,905,996 journeys. Given further information, it would be desirable to treat the length of disruption as a stochastic variable.

Since failure probabilities are very small, it is assumed that flood events and their impacts are independent and that the disruption from different flood events is additive. With flood events simulated at a mean rate of 4.3 per year, the annualized expected disruption is estimated to be 1,905,996 × 4.3 = 8,195,783 journeys.

This represents 0.5% of the mean annual passenger flow of 1.7 × 10^9^ journeys per year (HM Government, 2017a) on the British rail network. Recent economic analysis places the network's economic benefit to passengers between £1.2 billion and £12 billion per year (Oxera, 2014). The annual cost to passengers of the loss of 0.5% network utilization can therefore be valued at between £6 million and £60 million.

### 7.2 Safety Risk

Safety has long been a paramount concern for Britain's railways, beginning in 1840 with the establishment of a Railway Inspectorate and a requirement to report all injurious accidents (Hutter, 2001). Today, safety risk management follows principles set out in the common safety method for risk evaluation and assessment (CSM RA) (Office of Rail Regulation, 2015a). One or more of the following three risk acceptance principles can be applied: (1) application of codes of practice, (2) comparison with similar reference systems, or (3) explicit risk estimation. We can compare our analysis of economic disruption from scour with the Railway Safety and Standards Board Safety Risk Model (SRM) (Rail Safety and Standards Board, 2018), which quantifies safety risk in units of fatalities and weighted injuries (FWIs) (Rail Safety and Standards Board, 2008).

The SRM (v8.5, March 2018) estimates the frequency of structural collapse due to scour and water action leading to passenger train derailments to be 3.62 × 10^−3^ events per year, leading to a risk of 9.43 × 10^−3^ FWIs per year (we focus on passenger trains, excluding freight, to compare with our economic analysis of loss of utility to passengers). The economic value of preventing a statistical fatality adopted by the industry is approximately £1.9 million (Rail Safety and Standards Board, 2017), which means that the annual safety risk associated with derailments of passenger trains, when quantified in the same economic terms, is less than £20,000. With safety being a fundamental driver for the industry (Office of Rail Regulation, 2015b), our analysis suggests that loss of utility to passengers from the residual bridge scour risk has orders of magnitude greater economic impact than the residual safety risk, which has been significantly mitigated.

It should also be recognized that safety and economic concerns motivate management practices in different ways. Safety concerns are paramount, motivating intensified surveillance, speed restrictions, and, indeed, line closures during extreme flood events. These practices incur some economic losses for passengers even in the absence of bridge failures, while meaning that the probability of fatal bridge failures is much lower than would otherwise be the case.

### 7.3 Future Applications of the Analysis

Economic assessment of safety risk can be a part of a cost–benefit analysis when determining whether a specific measure is necessary to ensure “safety so far as is reasonably practicable,” an important principle in the railway industry (Rail Safety and Standards Board, 2014). Passenger utility, our present focus, is considered within the industry's wider business planning. Our analysis demonstrates an approach for explicit risk estimation in this context, analogous to the third of the safety risk acceptance principles outlined above. We hope to support future debate about risk acceptance by adding this economic dimension, which was not previously available.

The framework demonstrated here can also support further analysis of the business case for investments in scour mitigation. The generic fragility model provides a baseline against which improvements in risk mitigation may be compared. At present, there is no model available to specify changes in the fragility function for specific scour mitigation measures, but an earlier expert elicitation study (Lamb et al., 2017) offers some evidence, for generic bridge and watercourse types, about how failure probabilities might change under three different sets of assumptions about scour risk mitigation actions. These compare a “business as usual” assumption with two counterfactuals: no scour mitigation, or significantly enhanced mitigation. The results showed order of magnitude changes in the experts’ judgments of failure probabilities, which could be implemented within our risk analysis to quantify the difference in risk, and hence economic benefits, of different levels of risk mitigation. Further research would be needed (see below) to link those changes to specific changes in scour management practices. An approach to testing alternative mitigation strategies has been demonstrated for the Chinese railway system (L. Hong, Ouyang, Peeta, He, & Yan, 2015) using a network risk model in which a scaling factor, representing a “maintenance intensity,” was applied to modify failure rates under stochastically generated event scenarios. A similar approach could be applied with our model.

By combining spatially coherent models for flood hazard and passenger journey disruption, our analysis could be adapted to study the contribution to risk from specific extreme event scenarios. We have shown that rare events in which multiple bridges fail contribute significantly to the total risk, which would be underestimated if a simpler model, lacking spatial dependence, were applied to represent the flood hazard.

Our model may also be applied to help the industry and emergency planners in preparing for the impacts and operational consequences of extreme flood scenarios through the development of “stress test” scenarios. In the United Kingdom, the government's National Risk Assessment (Cabinet Office, 2017) is the basis for assessing emergency preparedness against a set of hazards, specified as scenarios within defined probability and impact bounds. Probability bounds are set so as to provide scenarios that would be challenging for emergency responders, but not implausible. It is therefore important that the scenarios can be interpreted probabilistically. For inland flooding, the National Risk Assessment considers a probability of occurrence between 1/200 and 1/20 over five years, which was assessed using the same type of spatial flood hazard model as applied in this article (Wood et al., 2016). Our model could be used to develop similar scenarios relating to disruption to rail services caused by flooding and scour, with quantified probabilities and consequences.

Overall, this analysis provides the methodology and tools to undertake a first‐order screening whereby locations and assets causing high systemic risks can be prioritized for further detailed structural engineering investigations. In a large‐scale network, such information becomes very useful when there are limited budgets to invest in asset risk management.

### 7.4 Known Limitations and Opportunities for Further Research

A number of limitations are acknowledged in this analysis. Our empirical model does not represent scour processes physically and is dependent on the set of failure events that happen to have been observed. It does not account explicitly for temporal processes, especially sequences of events that may explain collapses in relatively minor floods. Similarly, some recent events are known to have involved blockage (e.g., the Feltham bridge failure in 2009; RAIB, 2009), which is not accounted for explicitly, though it is known that debris mobilization increases markedly in extreme floods (Comiti, Lucía, & Rickenmann, 2016; McIntyre & Thorne, 2013; Weeks, Witheridge, Rigby, Barthelmess, & O'Loughlin, 2013). Long‐term nonstationarity in the probability distribution of historic flood events is ignored, as are systematic changes in construction and maintenance standards, or repair and replacement of bridges. Additionally, variations in foundation types, construction dates, and watercourse typology are not accounted for, other than as a contribution to uncertainty in the fragility analysis. Our standardization methodology assumes that bridges have been built to withstand roughly the same return period flood irrespective of the mean flow in the river. Failure processes are assumed to be independent between bridges, given the flow conditions, meaning that we do not allow for a cascading failure mechanism, as could occur more obviously in situations such as dam breaches where an asset failure leads to the release of a large flood wave. We also assume that there are no systematic weaknesses in particular subsets of bridges, or at particular times, that might cause failures to cluster (other than through a common high level of loading simulated from the spatial flood hazard model).

The factors mentioned above are captured implicitly in the dispersion parameter of the fragility function and in the uncertainty analysis. With an understanding of its limitations, the fragility function is interpreted as a model for a broad‐scale risk analysis, that is, we consider the risk of scour at an aggregate level, over the whole rail network, but cannot attribute risk to individual bridges.

Although passenger journey disruption provides one perspective on scour risk, other loss functions could be applied within this framework to account for economic, operational, or safety impacts of scour risk. The wider economic impacts go beyond the utility value for rail passengers, and include impacts on freight, business interruption, and other indirect consequences. These impacts can be significant; for example, the economic cost of a coastal railway line collapse at Dawlish in 2015 was estimated to be about £2.1 billion (BBC, 2015).

Further research could add more granularity to the model proposed here by classifying failure events according to bridge typology or failure mechanism, albeit at the cost of reducing the effective sample size. As noted in Section 7.3, the fragility function could also be adjusted to reflect differing scenarios for construction, maintenance, or scour protection standards, and hence reflect different risk mitigation investment strategies. This will require research to determine changes in failure probability linked to specific changes in risk mitigation actions, and their associated costs. Furthermore, the loading model may be adjusted to reflect changes in hydrological regime, especially to allow climate change scenarios to be considered.

The integration of the fragility function with a spatial flood hazard model allows the likelihood of specific failure scenarios to be quantified. For example, certain bridges are more critical than others in terms of potential disruption (Pant, Blainey, Hall, & Preston, 2015), hence it may be useful to assess scenarios conditional on failure events at those locations. Although concurrent failures of multiple structures are very rare, they have occurred even in recent decades, hence are known to contribute to the network risk. A low‐probability, high‐consequence scenario of this type, such as failure of multiple bridges on major routes around London, could now be assessed within a probabilistic framework by applying the approach taken here.

## 8 CONCLUSIONS

Using a data set of 100 railway bridge failures dating from 1846, we have estimated a fragility curve for scour failure conditional upon the severity of flood events at a bridge. The method incorporates information from river flow records that coincided with bridge failures, where available, and censored data elsewhere, within an MLE framework. We estimate the conditional bridge failure probability to be 0.010 (range 0.002–0.02) in a 1/100 AEP flood event, and 0.062 (0.02–0.1) in a 1/1,000 AEP event.

We combined the scour fragility model with a statistical model for spatially coherent flood events to create a probabilistic bridge failure model. The known bridge failures are attributed to 54 historical flood events. In repeated samples of 54 events simulated from the linked flooding and fragility models, the average number of bridge failures is 1.97 (range 1.78–2.41), which compares well with the observed mean of 1.85 (range 1.34–2.38).

By integrating the probabilistic failure model with a network passenger journey model, we have quantified the risk of disruption due to scour over the British rail network. The annual risk is expressed as an average of 8.2 million disrupted passenger journeys. This estimate can be translated into an expected annual utility cost to passengers of between £6 million and £60 million. This estimate includes important contributions (76%) from low‐probability, high‐consequence scenarios containing multiple bridge failures, which can only be captured by modeling both flood hazard and bridge failures spatially, as we have done here. The loss of utility to passengers is merely one element of the wider costs of (rail) bridge scour risk to the U.K. economy. The costs associated with disruption to rail freight, spending on bridge repairs, delays caused by speed restrictions (imposed when scour damage is suspected), and injury or loss of life have not been computed, but could be accounted for in the same framework if suitable models are developed to quantify them.

The statistical approach proposed here represents an integrated, network‐scale risk assessment conditioned on historical observations of river flooding and bridge failures. The same framework could be generalized for other weather‐related hazards, asset types, and failure modes, such as extreme rainfall and earthworks failures, or for other infrastructure networks. Though the inclusion of greater site‐specific information on bridge characteristics and vulnerability is attractive in principle, in practice records of observed failures (and the conditions associated with those failures) are very limited, so empirical methods of the type proposed here are bound to be more generic. Appropriate levels of investment in risk mitigation are not only determined by the scale of the risk, but by the sensitivity of reductions in risk, that is, the marginal benefits, achieved with different mitigation strategies. This article has demonstrated the integration of generic fragility curves within a probabilistic risk assessment framework that has not hitherto been feasible, and that could be applied in future to investigate those benefits of risk mitigation.

**NOMENCLATURE**
*Y*: random variable describing uncertain loading condition, *y*, in fragility analysis
*g_Y_*(*y*), *G_Y_*(*y*): pdf and cdf of load variable
*F_Y_*(*y*): fragility function
*s*: state variable describing whether a bridge has failed, or a network edge has incurred one or more bridge failures
*z*(*s*): damages incurred for a given failure state
*μ*: expected damages
*λ*: expected number of bridge failures
Δ: a set containing all bridges in the rail network
Λ: a set containing all edges in the rail network topology
*X*: random variable describing peak river flow rates, *x*
*τ*: return period of a flood event
*γ*: ratio between historical mean and contemporary railway network lengths
Ψ: subset of potential network failure states
*θ*, *β*: parameters of fragility function
*ϕ*, Φ: standard normal probability density function and distribution function, respectively
*χ*^2^: chi‐squared distribution

## References

[risa13370-bib-0001] Association of Train Operating Companies . (2013a). *Passenger demand forecasting handbook* (version 5.1). Retrieved from https://www.raildeliverygroup.com/pdfc.html.

[risa13370-bib-0002] Association of Train Operating Companies . (2013b). *Timetable data* . Retrieved from http://data.atoc.org.

[risa13370-bib-0003] BBC . (2015). Dawlish rail line: Closure “costs economy up to £1.2 bn.” *News* Retrieved from http://www.bbc.co.uk/news/uk-england-devon-31140192.

[risa13370-bib-0004] Black, A. R. , & Law, F. M. (2004). Development and utilization of a national web‐based chronology of hydrological events. Hydrological Sciences Journal, 49(2). 10.1623/hysj.49.2.237.34835

[risa13370-bib-0005] Black, A. R. , & Werritty, A. (1997). Seasonality of flooding: A case study of North Britain. Journal of Hydrology, 195(1–4), 1–25. 10.1016/s0022-1694(96)03264-7

[risa13370-bib-0006] Buijs, F. , Simm, J. , Wallis, M. , & Sayers, P. (2007). *Performance and reliability of flood and coastal defences* (FD2318/TR1). Retrieved from http://randd.defra.gov.uk/Default.aspx?Module=More&Location=None&ProjectID=11615.

[risa13370-bib-0007] Cabinet Office . (2017). *National risk register of civil emergencies* (2017 edition). Retrieved from https://www.gov.uk/government/publications/national-risk-register-of-civil-emergencies-2017-edition.

[risa13370-bib-0008] Centre for Ecology and Hydrology . (2017). *National river flow archive* . Retrieved from http://nrfa.ceh.ac.uk/.

[risa13370-bib-0009] Clarke, R. (1994). Statistical modelling in hydrology. Chichester: Wiley.

[risa13370-bib-0010] Coleman, S. E. , Lauchlan, C. S. , & Melville, B. W. (2003). Clear‐water scour development at bridge abutments. Journal of Hydraulic Research, 41(5), 521–531. 10.1080/00221680309499997

[risa13370-bib-0011] Comiti, F. , Lucía, A. , & Rickenmann, D. (2016). Large wood recruitment and transport during large floods: A review. Geomorphology, 269, 23–39. 10.1016/j.geomorph.2016.06.016

[risa13370-bib-0012] Cumbria County Council . (2017). *Bridges and roads update* . Retrieved from https://www.cumbria.gov.uk/eLibrary/Content/Internet/536/6181/42769141953.pdf.

[risa13370-bib-0013] Cunderlik, J. M. , Ouarda, T. B. M. J. , & Bobée, B. (2004). On the objective identification of flood seasons. Water Resources Research, 40(1). 10.1029/2003wr002295

[risa13370-bib-0014] Decò, A. , & Frangopol, D. M. (2011). Risk assessment of highway bridges under multiple hazards. Journal of Risk Research, 14(9), 1057–1089. 10.1080/13669877.2011.571789

[risa13370-bib-0015] Environment Agency . (2011). *The risk of widespread flooding: Capturing spatial patterns in flood risk from rivers and coasts (Project SC060088)* (978‐1‐84911‐247‐5). Retrieved from https://www.gov.uk/government/publications/the-risk-of-widespread-flooding-capturing-spatial-patterns-in-flood-risk-from-rivers-and-coasts.

[risa13370-bib-0016] Federal Emergency Management Agency . (2012). *Seismic performance assessment of buildings* (Vol. 1: Methodology, P‐58‐1). Retrieved from https://www.fema.gov/media-library/assets/documents/90380.

[risa13370-bib-0017] Federal Highway Administration . (2017a). *National bridge inspection standards* . Retrieved from https://www.fhwa.dot.gov/bridge/nbis.cfm.

[risa13370-bib-0018] Federal Highway Administration . (2017b). *National bridge inventory (NBI)* . Retrieved from https://www.fhwa.dot.gov/bridge/nbi.cfm.

[risa13370-bib-0019] Flint, M. M. , Fringer, O. , Billington, S. L. , Freyberg, D. , & Diffenbaugh, N. S. (2017). Historical analysis of hydraulic bridge collapses in the continental United States. Journal of Infrastructure Systems, 23(3). 10.1061/(asce)is.1943-555x.0000354

[risa13370-bib-0020] Hall, J. , & Blöschl, G. (2018). Spatial patterns and characteristics of flood seasonality in Europe. Hydrology and Earth System Sciences, 22, 3883–3901. 10.5194/hess-22-3883-2018

[risa13370-bib-0021] Hall, J. W. , Dawson, R. J. , Sayers, P. B. , Rosu, C. , Chatterton, J. B. , & Deakin, R. (2003). A methodology for national‐scale flood risk assessment. Proceedings of the Institution of Civil Engineers—Water and Maritime Engineering, 156(3), 235–247. 10.1680/wame.2003.156.3.235

[risa13370-bib-0022] Haywood, R. (2007). Britain's national railway network: Fit for purpose in the 21st century? Journal of Transport Geography, 15(3), 198–216. 10.1016/j.jtrangeo.2006.02.015

[risa13370-bib-0023] Heffernan, J. E. , & Tawn, J. A. (2004). A conditional approach for multivariate extreme values (with discussion). Journal of the Royal Statistical Society: Series B (Statistical Methodology), 66(3), 497–546. 10.1111/j.1467-9868.2004.02050.x

[risa13370-bib-0024] HM Government . (2016). *National flood resilience review* . Retrieved from https://www.gov.uk/government/publications/national-flood-resilience-review.

[risa13370-bib-0025] HM Government . (2017a). *Rail trends factsheet, 2016* . *Official statistics* Retrieved from https://www.gov.uk/government/statistics/rail-factsheets-2016.

[risa13370-bib-0026] HM Government . (2017b). *Table RAI0101, length of national railway route at year end, and passenger travel by national railway and London Underground* . Retrieved from https://www.gov.uk/government/statistical-data-sets/rai01-length-of-route-distance-travelled-age-of-stock.

[risa13370-bib-0027] Hong, J. H. , Goyal, M. K. , Chiew, Y. M. , & Chua, L. H. C. (2012). Predicting time‐dependent pier scour depth with support vector regression. Journal of Hydrology, 468–469, 241–248. 10.1016/j.jhydrol.2012.08.038

[risa13370-bib-0028] Hong, L. , Ouyang, M. , Peeta, S. , He, X. Z. , & Yan, Y. Z. (2015). Vulnerability assessment and mitigation for the Chinese railway system under floods. Reliability Engineering & System Safety, 137, 58–68. 10.1016/j.ress.2014.12.013

[risa13370-bib-0029] Hutter, B. M. (2001). The railway inspectorate: Regulatory objectives and the social dimensions of knowledge (Regulation and risk—Occupational health and safety on the railway). Oxford: Oxford University Press.

[risa13370-bib-0030] Kattell, J. , & Eriksson, M. (1998). *Bridge scour evaluation: Screening, analysis and countermeasures* (9877 1207–SDTDC). Retrieved from https://www.fs.fed.us/eng/structures/98771207.pdf.

[risa13370-bib-0031] Keef, C. , Tawn, J. A. , & Lamb, R. (2013). Estimating the probability of widespread flood events. Environmetrics, 24(1), 13–21. 10.1002/env.2190

[risa13370-bib-0032] Kim, S.‐H. , & Feng, M. Q. (2003). Fragility analysis of bridges under ground motion with spatial variation. International Journal of Non‐Linear Mechanics, 38(5), 705–721. 10.1016/s0020-7462(01)00128-7

[risa13370-bib-0033] Kirby, A. M. , Roca, M. , Kitchen, A. , Escarameia, M. , & Chesterton, O. J. (2017). Manual on scour at bridges and other hydraulic structures (2nd ed., Vol. C742). London: CIRIA.

[risa13370-bib-0034] Kjeldsen, T. R. , & Jones, D. A. (2010). Predicting the index flood in ungauged UK catchments: On the link between data‐transfer and spatial model error structure. Journal of Hydrology, 387(1–2), 1–9. 10.1016/j.jhydrol.2010.03.024

[risa13370-bib-0035] Kjeldsen, T. R. , Jones, D. A. , & Morris, D. G. (2014). Using multiple donor sites for enhanced flood estimation in ungauged catchments. Water Resources Research, 50(8), 6646–6657. 10.1002/2013wr015203

[risa13370-bib-0036] Klugman, S. A. , Panjer, H. H. , & Willmot, G. E. (2004). Loss models: From data to decisions (2nd ed.). Hoboken, NJ: Wiley.

[risa13370-bib-0037] Lamb, R. , Aspinall, W. , Odbert, H. , & Wagener, T. (2017). Vulnerability of bridges to scour: Insights from an international expert elicitation workshop. Natural Hazards and Earth System Sciences, 17(8), 1393–1409. 10.5194/nhess-17-1393-2017

[risa13370-bib-0038] Lamb, R. , Keef, C. , Tawn, J. , Laeger, S. , Meadowcroft, I. , Surendran, S. , *…* Batstone, C. (2010). A new method to assess the risk of local and widespread flooding on rivers and coasts. Journal of Flood Risk Management, 3(4), 323–336. 10.1111/j.1753-318X.2010.01081.x

[risa13370-bib-0039] Martí‐Henneberg, J. (2013). European integration and national models for railway networks (1840–2010). Journal of Transport Geography, 26, 126–138. 10.1016/j.jtrangeo.2012.09.004

[risa13370-bib-0040] McCullagh, P. , & Nelder, J. A. (1989). Generalized linear models (2nd ed.). Boca Raton, FL: Chapman & Hall/CRC Press.

[risa13370-bib-0041] McIntyre N., & Thorne C. (Eds.). (2013). C719—Land use management effects on flood flows and sediments. London: CIRIA.

[risa13370-bib-0042] Melville, B. W. (1997). Pier and abutment scour: Integrated approach. Journal of Hydraulic Engineering‐ASCE, 123(2), 125–136. 10.1061/(Asce)0733-9429(1997)123:2(125)

[risa13370-bib-0043] Melville, B. W. , & Chiew, Y. M. (1999). Time scale for local scour at bridge piers. Journal of Hydraulic Engineering‐ASCE, 125(1), 59–65. 10.1061/(Asce)0733-9429(1999)125:1(59)

[risa13370-bib-0044] Nelder, J. A. , & Mead, R. (1965). A simplex method for function minimization. Computer Journal, 7(4), 308–313. 10.1093/comjnl/7.4.308

[risa13370-bib-0045] Office of Rail Regulation . (2013). *Estimates of station usage* . Retrieved from http://www.rail-reg.gov.uk/server/show/nav.1529.

[risa13370-bib-0046] Office of Rail Regulation . (2015a). *Common safety method for risk evaluation and assessment: Guidance on the application of Commission Regulation (EU) 402/2013* . Retrieved from http://orr.gov.uk/__data/assets/pdf_file/0006/3867/common_safety_method_guidance.pdf.

[risa13370-bib-0047] Office of Rail Regulation . (2015b). *ORR's health and safety regulatory strategy* . Retrieved from http://orr.gov.uk/__data/assets/pdf_file/0018/17019/health-and-safety-regulatory-strategy.pdf.

[risa13370-bib-0048] Oxera . (2014). *What is the contribution of rail to the UK economy* ? Retrieved from https://www.oxera.com/Latest-Thinking/Publications/Reports/2014/What-is-the-contribution-of-rail-to-the-UK-economy.aspx.

[risa13370-bib-0049] Pant, R. , Blainey, S. , Hall, J. , & Preston, J. (2015). *Assessing risks to inform resilience: A criticality assessment of the British railway network* . Paper presented at the International Symposium for Next Generation Infrastructure (ISNGI 2014), International Institute of Applied Systems Analysis (IIASA), Schloss Laxenburg, Vienna, Austria. Retrieved from http://discovery.ucl.ac.uk/id/eprint/1469293.

[risa13370-bib-0050] Pant, R. , Hall, J. W. , & Blainey, S. P. (2016). Vulnerability assessment framework for interdependent critical infrastructures: Case‐study for Great Britain's rail network. European Journal of Transport and Infrastructure Research, 16(1), 174–194.

[risa13370-bib-0051] Porter, K. , Kennedy, R. , & Bachman, R. (2007). Creating fragility functions for performance‐based earthquake engineering. Earthquake Spectra, 23(2), 471–489. 10.1193/1.2720892

[risa13370-bib-0052] RAIB . (2009). *Failure of Bridge RDG1 48 (River Crane) between Whitton and Feltham* (Report 17/2010). Retrieved from https://assets.publishing.service.gov.uk/media/547c8ff7e5274a4290000195/R172010_100923_Feltham.pdf.

[risa13370-bib-0053] Rail Safety and Standards Board . (2004). *Impact of scour and flood risk on railway structures* (T112). Retrieved from https://www.rssb.co.uk/research-development-and-innovation/research-and-development/research-project-catalogue/t112.

[risa13370-bib-0054] Rail Safety and Standards Board . (2008). *The weighting of non‐fatal injuries. Fatalities and weighted injuries* (T440). Retrieved from https://www.rssb.co.uk/research-development-and-innovation/research-reports-catalogue/pb009573.

[risa13370-bib-0055] Rail Safety and Standards Board . (2014). *Guidance on the use of cost‐benefit analysis when determining whether a measure is necessary to ensure safety so far as is reasonably practicable* . Retrieved from https://www.rssb.co.uk/Library/risk-analysis-and-safety-reporting/2014-guidance-safety-related-cba.pdf.

[risa13370-bib-0056] Rail Safety and Standards Board . (2017). *Taking safe decisions—Safety‐related CBA* . Retrieved from https://www.rssb.co.uk/risk-analysis-and-safety-reporting/risk-analysis/taking-safe-decisions/taking-safe-decisions-safety-related-cba.

[risa13370-bib-0057] Rail Safety and Standards Board . (2018). *Safety risk model, version 8.5* . Retrieved from https://www.rssb.co.uk/safety-risk-model/risk-profile-bulletin/Documents/Safety%20Risk%20Model%20v8.5.0%20-%20Overview.pdf.

[risa13370-bib-0058] Shinozuka, M. , Feng, M. Q. , Lee, J. , & Naganuma, T. (2000). Statistical analysis of fragility curves. Journal of Engineering Mechanics, 126(12), 1224–1231. 10.1061/(asce)0733-9399(2000)126:12(1224)

[risa13370-bib-0059] Stein, S. M. , Young, G. K. , Trent, R. E. , & Pearson, D. R. (1999). Prioritizing scour vulnerable bridges using risk. Journal of Infrastructure Systems, 5(3), 95–101. 10.1061/(asce)1076-0342(1999)5:3(95)

[risa13370-bib-0060] UK Roads Liaison Group . (2009). *Background briefing on highway bridges* . Retrieved from http://www.ukroadsliaisongroup.org/en/utilities/document-summary.cfm?docid=59ABF16C-03DE-4864-B31C64A86371A89F.

[risa13370-bib-0061] US Army Corps of Engineers . (1993). *Reliability assessment of existing levees for benefit determination* (Engineer Technical Letter 1110‐2‐328), Washington, DC. Retrieved from https://www.publications.usace.army.mil/USACE-Publications/Engineer-Technical-Letters/.

[risa13370-bib-0062] van Erp, H. R. N. , Linger, R. , Khakzad, N. , & van Gelder, P. (2017). *Report on risk analysis framework for collateral impacts of cascading effects* (D 5.2, RAIN—Risk Analysis of Infrastructure Networks in Response to Extreme Weather). Retrieved from http://rain-project.eu/wp-content/uploads/2017/08/D5_2_Final_merged.pdf.

[risa13370-bib-0063] Van Gelder, P. , Buijs, F. , Van, C. M. , Horst, W. , Kanning, W. , Nejad, M. , *…* Lambrecht, H.‐J. (2008). *Reliability analysis of flood and sea defence structures and systems* (T07‐08‐01). Retrieved from http://www.floodsite.net/html/partner_area/project_docs/T07_08_01_Reliability_Analysis_D7_1.pdf.

[risa13370-bib-0064] van Leeuwen, Z. , & Lamb, R. (2014). *Flood and scour related failure incidents at railway assets between 1846 and 2013* (W13‐4224). Retrieved from http://www.jbatrust.org/wp-content/uploads/2016/01/JBA-Trust-Flood-and-scour-failure-at-railway-assets-1846-to-2013-W13-4224-FINAL.pdf.

[risa13370-bib-0065] Weeks, W. , Witheridge, W. , Rigby, G. , Barthelmess, E. A. , & O'Loughlin, G. (2013). *Australian Rainfall and Runoff, Project 11: Blockage of hydraulic structures* (P11/S2/021). Retrieved from http://arr.ga.gov.au/__data/assets/pdf_file/0008/40499/ARR_Project_11_Stage2_report_Final.pdf.

[risa13370-bib-0066] Whitbread, J. E. , Benn, J. R. , & Hailes, J. M. (2000). Cost‐effective management of scour‐prone bridges. Proceedings of the Institution of Civil Engineers—Transport, 141(2), 79–86. 10.1680/tran.2000.141.2.79

[risa13370-bib-0067] Wood, E. , Lamb, R. , Warren, S. , Hunter, N. , Tawn, J. , Allan, R. , & Laeger, S. (2016). Development of large scale inland flood scenarios for disaster response planning based on spatial/temporal conditional probability analysis. E3S Web of Conferences, 7, 10.1051/e3sconf/20160701003

[risa13370-bib-0068] Zevenbergen, L. W. (2010). Comparison of the HEC‐18, Melville and Sheppard pier scour equations. Geotechnical Special Publication, 210, 1074–1081. 10.1061/41147(392)108

